# Reply: Comment on ‘Developing and paying for medicines for orphan indications in oncology: utilitarian regulation *vs* equitable care?’

**DOI:** 10.1038/bjc.2012.281

**Published:** 2012-06-28

**Authors:** J E Davies, S Neidle, D G Taylor

**Affiliations:** 1 Department of Practice and Policy, School of Pharmacy, BMA/Tavistock House, London WC1H 9JP, UK; 2 Department of Chemical Biology and Centre for Cancer Medicines, School of Pharmacy, University of London, London, UK

**Sir**,

We note Professor Bielack’s concern (Bielack, 2012) regarding our reference to his *European Journal of Cancer Care* editorial ‘Osteosarcoma: time to move on?’ (Bielack, 2010). However, he misinterprets the reason for our reference to his contribution. We were not suggesting that Professor Bielack had said that he had fears that ‘the viability of the EURAMOS trial of established therapies would be impaired if more osteosarcoma patients were able to access MTP’.

Rather, we referred to his editorial primarily because it describes the scale of the EURAMOS trial, and secondarily because it illustrates aspects of the debate surrounding the value of MTP. In the latter context, we note that in the United Kingdom, towards the end of 2011, NICE recommended MTP as cost-effective for NHS use in the treatment of osteosarcoma, albeit this was some 30 months after this medicine was licensed by the EMEA.

We wish to emphasise that the purpose of our article (Davies *et al*, 2012) was to highlight the difficulties that innovators may face when they seek to have medicines for orphan and ultra-orphan indications licensed, and made available for use on a reimbursed basis. The MPT example illustrates a range of relevant barriers and inconsistencies, but it is not in itself central to our argument.
